# Structure Evolution of Ordered Mesoporous Carbons Induced by Water Content of Mixed Solvents Water/Ethanol

**DOI:** 10.1186/s11671-016-1569-4

**Published:** 2016-08-12

**Authors:** Peng Li, Shujun Liang, Zhenzhong Li, Yan Zhai, Yan Song

**Affiliations:** 1 Department of Materials Engineering, Taiyuan Institute of Technology, Taiyuan, 030008 China; 2 Key Laboratory of Carbon Materials, Institute of Coal Chemistry, Taiyuan, 030001 China

**Keywords:** Structure evolution, Ordered mesoporous carbons, Soft template, Cosolvent

## Abstract

In this work, mesostructure evolution of ordered mesoporous carbons (OMCs) from the 2-D hexagonal (space group p6mm) to the discontinuous cubic $\mathrm{F}\mathrm{d}\overline{3}\mathrm{m}$, then towards the face-centered cubic lattice $\mathrm{F}\mathrm{m}\overline{3}\mathrm{m}$, and finally, to the simple cubic Pm3n is achieved by simply adjusting the cosolvent water content of the mixed solvents water/ethanol in the presence of a reverse nonionic triblock copolymer and low molecular resin by evaporation-induced self-assembly method. Experimental results demonstrate that both the cosolvent and the reverse triblock copolymer play a key role in the mesophase transitions of OMCs. Furthermore, the OMCs with Pm3n symmetry are reported for the first time. Finally, the mechanism of mesostructure transition was discussed and proposed.

**Electronic supplementary material:**

The online version of this article (doi:10.1186/s11671-016-1569-4) contains supplementary material, which is available to authorized users.

## Background

Ordered mesoporous carbons (OMCs) have been studied extensively since their discovery focusing on their synthesis control as well as their applications such as catalyst support [1], electrodes materials [2], and delivering processing [3]. Over the past years, most synthesis strategies of OMCs are related to hard template using technique such as silica or colloids which might provide precise control in pore structure, high surface areas, and great pore volumes [4]. However, it is unavoidable to use toxic chemicals such as hydrofluoric acid to remove the templates. Since Dai et al. first got OMCs through the organic-organic self-assembly method, which only needs the nonionic block copolymer as a structure-directing agent [2]. Several groups have concentrated on this simple method. For example, Tanaka et al. synthesized the OMCs with a hexagonal channel structure using RF and triethyl orthoacetate (EOA) in acidic condition. The author points out that EOA plays an important role in stabling the ordered mesostructure [5]. More recently, Zhao’s group developed a powerful method to prepare OMCs directly from low-molecular-weight phenolic resol precursors and a triblock copolymer or several mixed triblock copolymers via a solvent evaporation-induced self-assembly (EISA) method [6]. Liu et al. reported the direct synthesis of high OMCs through RF carbon precursor and F108 as a structure-directing agent [7]. During the past years, various mesostructures of OMCs including p6mm [8, 9], Im3m [7, 10, 11], Ia3d [12], $\mathrm{F}\mathrm{m}\overline{3}\mathrm{m}$ [13, 14], and $\mathrm{F}\mathrm{d}\overline{3}\mathrm{m}$ [15–17] symmetries have been synthesized by the soft template strategy with nonic copolymers as a structure-directing agent though carefully selecting the amphiphilic triblock copolymer.

Mesostructure control is a very exciting and challenging work in the synthesis of ordered mesoporous materials both in the academic and industrial areas. As it is well known, the type of solvent, the hydrolysis ratio, the concentration of surfactant, and carbon precursors are important factors for the EISA approach. Lots of effects on the formation of ordered mesoporous silica have been studied extensively such as by changing the counter anion [18–21] or by the variation of the solvent [22]. However, only very few papers have been done in the transformation of mesostructure of OMCs [23, 24]. Until very recently, we reported an intergrown p6mm and cubic Fd3m of OMCs induced by various ratios of ethanol/hexane [25]. Therefore, more work should be done on the factors that affect the mesophase of OMCs.

Furthermore, as one of the most interesting, peculiar 3-D pore structures, the Pm3n symmetry with two types of pores of OMCs is not reported until now probably because of the high curvature of its mesophase. In addition, up to now, there are few paper which report the ordered mesoporous silica with Pm3n symmetry using reverse triblock copolymer as a structure-directing agent though several groups have obtained this unique structure with gemini surfactants under acidic or basic condition [26, 27].

**Fig. 1:**
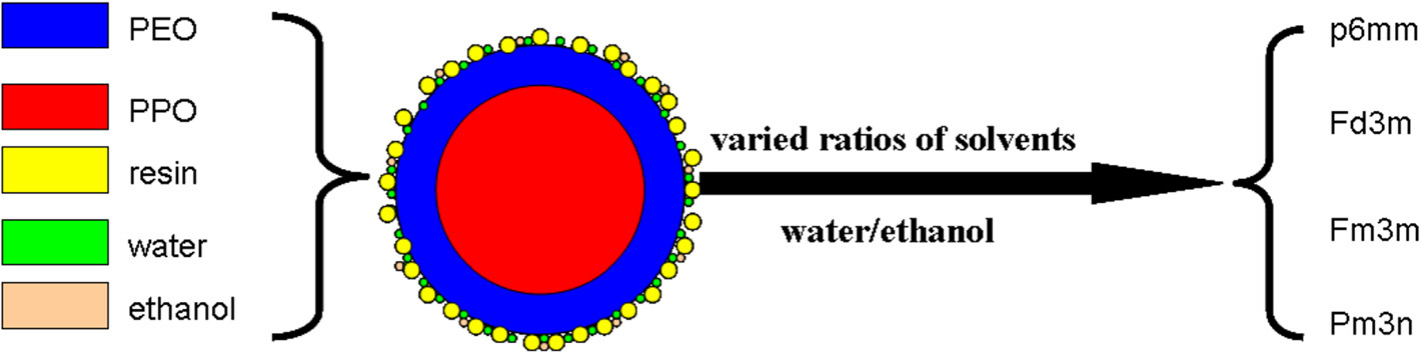
Schematic representation of mesostructure evolution induced by varied contents of cosolvent water of mixed water/ethanol

It was reported that some bounded water will participate in the formation of mesophase copolymer, i.e., the copolymer can form micelle which consists of a hydrophobic core composed mainly of polypropylene oxide (PPO) core and an out layer composed of a mixture of polyethylene oxide (PEO) and water [28–30]. It was concluded that if lots of water are present, water is not evenly distributed and the environments of EO units along PEO chains could be different. Herein, our goal has been to develop a novel method to control the mesostructure of OMCs through changing the content of the cosolvent water (Fig. 1). To the best of our knowledge, the facile mesostructure evolution of OMCs was reported for the first time by simply adjusting the water content of mixed solvents water/ethanol which will lead to the different curvatures of mesophase and synthesis of OMCs with cubic Pm3n symmetry using the home-made reverse triblock copolymer with long hydrophilic chain PEO. (PO_97_EO_186_PO_97_, MW = 19,500, purchased from Nanjing Chemical Corp.). Its molecular structure was confirmed by 1H NMR spectroscopy (ESI† Additional file 1: Figure S1), and it has a dispersive value of 1.35 by gel permeation chromatography (GPC).

## Methods

### Chemicals

Poly(propylene oxide)-poly(ethylene oxide)-poly(propylene oxide) triblock copolymer R (PO_97_EO_186_PO_97_, MW = 19,500) was purchased from by Nanjing Chemical Corp. F127 (EO_106_PO_70_EO_106_, MW = 12,600) was purchased from BASF Corp. Phenol, formalin solution (37–40 wt%), NaOH, and ethanol were purchased from Tianjin Chemical Corp. All chemicals were used as received without further purification.

### Experimental

Typically, 1.0 g of copolymer was dissolved in (20 − *X*) ml ethanol and *X* water mixed solvents under magnetic stirring at 30 °C. The volume of water, *X*, was in a certain range (*X* = 5, 6, 7, 8, 9, 10, 11, 12, 13, 14, 15 ml) to study the influence of water on the final mesostructures. Then, low-molecular-weight phenolic resol precursors were prepared as previously reported with some modifications [17]. The typical process was as follows: 1.0 g of phenol was melted at 45 °C before 0.31 g NaOH aqueous (20 wt%) was added into with stirring. 2.1 g of formalin (37–40 wt%) was added dropwise, and the mixture was stirred at 75 °C for 60 min. After cooling the mixture to room temperature, the pH of the reaction was adjusted to neutral (7.0) using 2.0 M HCl solution. Water was then removed under vacuum below 50 °C for 2 h. The final product was redissolved in ethanol and was added dropwise to the above solution containing copolymer, further stirred for 10 min. The solution was transferred to a dish, and the mixed solvents evaporated at room temperature over 8 h to produce a transparent membrane. The membrane was cured at 100 °C for 24 h in air for further thermopolymerization. The product was carbonized at 800 °C for 2 h, with a heating rate of 1 °C min^−1^ under nitrogen atmosphere; the obtained samples were abbreviated as OMCs−*X* (*X* = 5, 6, 7, 8, 9, 10, 11, 12, 13, 14, and 15, respectively). The samples were characterized by small-angle X-ray scattering (SAXS) recorded by using an imaging plate with X-ray wavelength of *λ* = 1.54 Å at beam line 4B9A 1W2A SAXS station of the Beijing Synchrotron Radiation Facility (BSRF). The data processing using the programs implies acceptance of acknowledging author Zhihong Li, transmission electron microscopy (TEM; F30 and Hitachi H-800 operated at 200 Kv). For the TEM measurements, OMC samples were crushed in an agate mortar, dispersed in ethanol, and deposited on a microgrid. N_2_ sorption techniques (Tristar 3000 analyzer at 77 K). Before the measurements of the sorption isotherms, the samples were outgassed at 200 °C in vacuum for 6 h.

## Results and Discussion

**Fig. 2:**
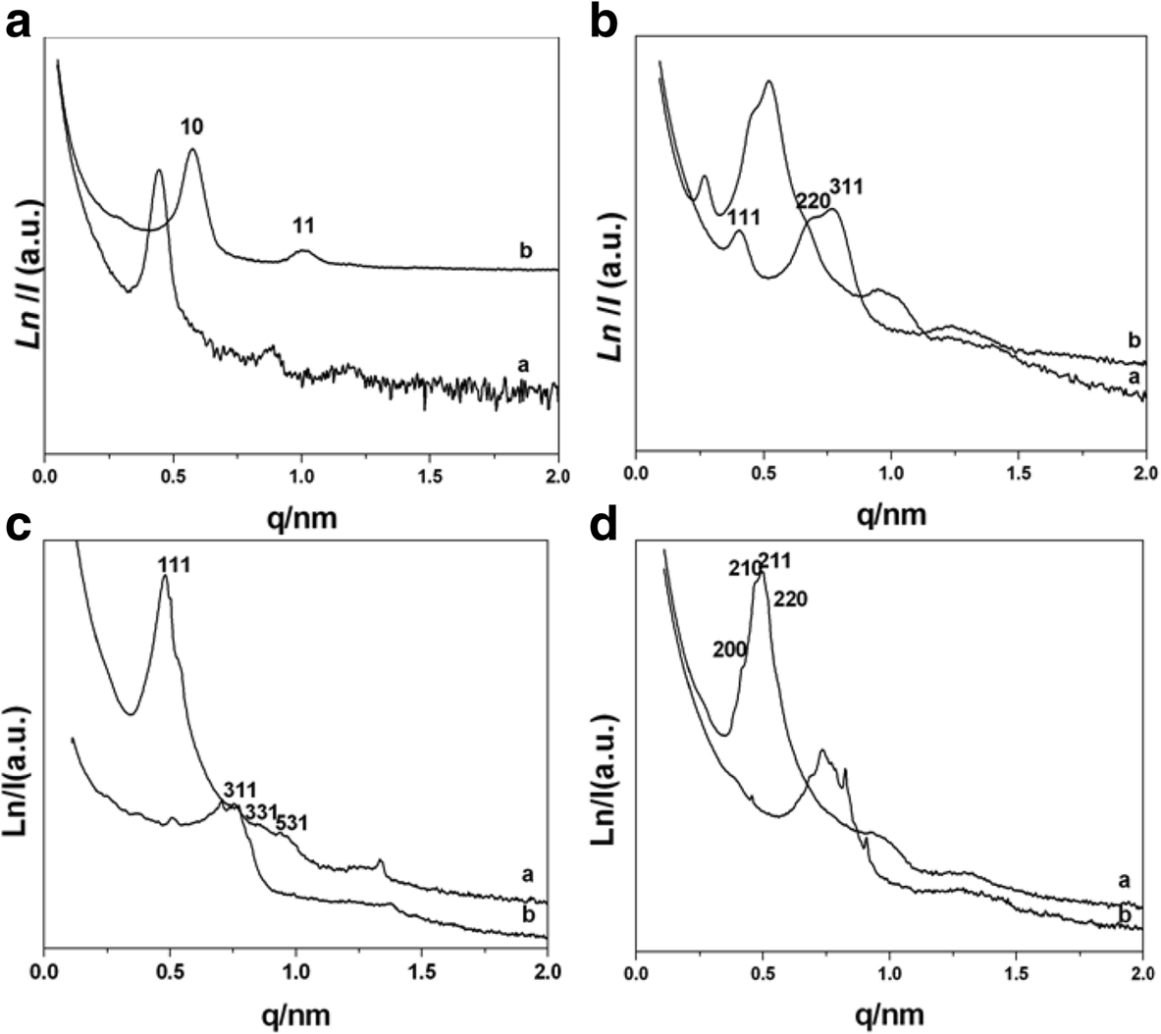
SAXS patterns of samples before (*a*) and after (*b*) calcinations with varied ratios of **a** 5/15, **b** 7/13, **c** 11/9, and **d** 12/8

**Fig. 3:**
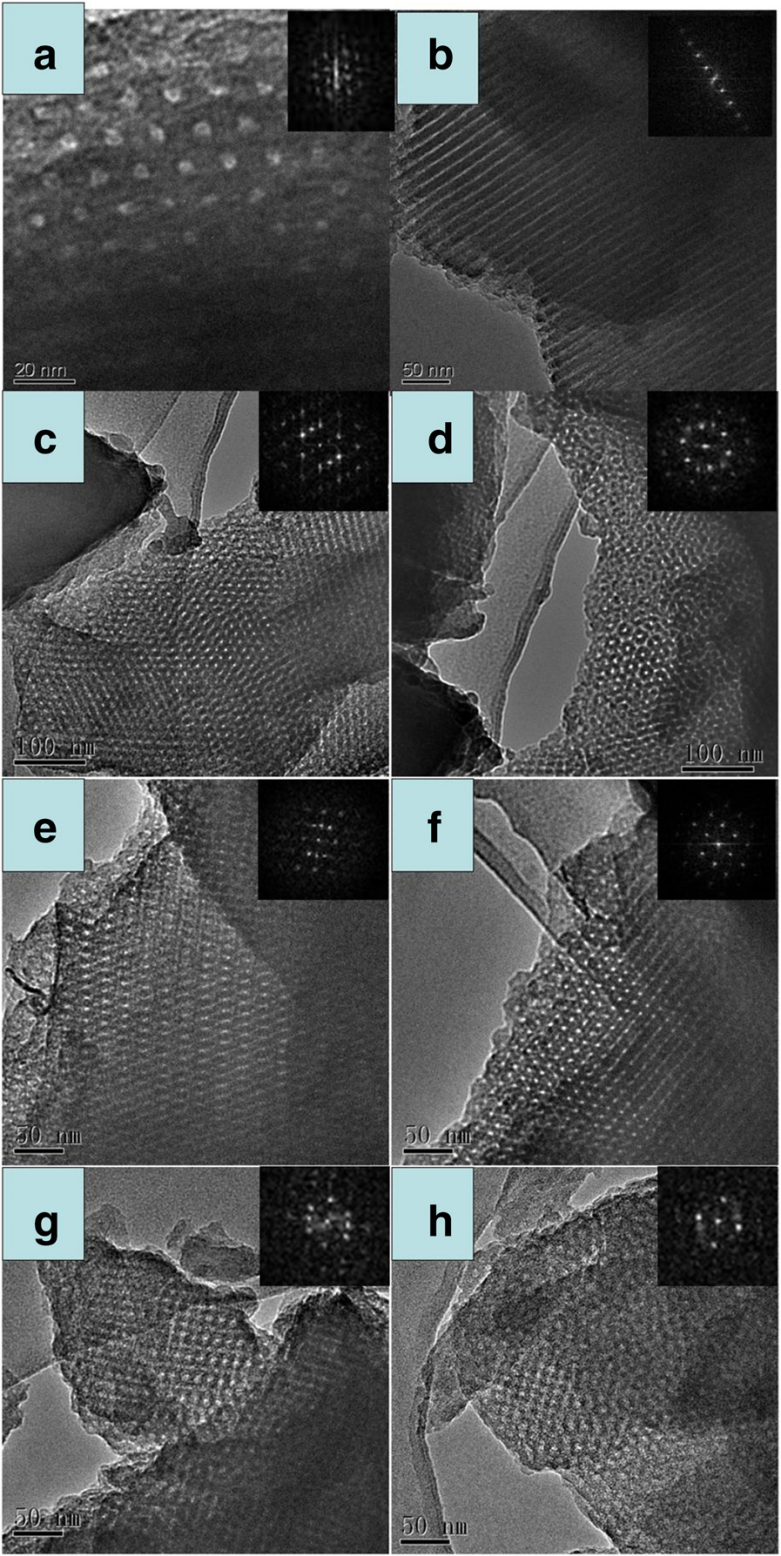
TEM images of OMCs-5, viewed from [100] (**a**) and [110] (**b**) directions of hexagonal structure. TEM images of OMCs-7 along the **c** [210] and **d** [110] directions and **e** [111] and **f** [210] directions of OMCs-11. **g** TEM images of OMCs-12 along the [211] and **h** [100] directions

Figure 2a shows the SAXS patterns of the sample before and after calcinations in the presence of ratio 5/15. The as-made sample before calcinations shows three well-resolved peaks (Fig. 2a (a)). They can be indexed to be [10], [11], and [20] planes of a 2-D hexagonal mesostructure (space group p6m), which is highly ordered [12]. Upon calcinations, the calcined sample gives two well-resolved peaks shown in Fig. 2a (b), implying that OMCs-5 is thermally stable. The *q* vectors move to higher values after calcinations, suggesting a contraction of the carbon framework. Representative hexagonally arranged and stripe-like TEM images of OMCs-5 with long-range order are visible along the [10] and [11] reflections of hexagonal phase, further confirming ordered 2-D hexagonal p6m mesostructure in Fig. 3a, b.

The SAXS patterns of the samples before and after calcinations synthesized in the ratio of 7/13 are shown in Fig. 2b. The uncalcined sample exhibits an unusual pattern (Fig. 2b (b)). At least three resolved diffraction peaks can be observed at *q* values of 0.2–0.9 nm^−1^. The lattice parameter (*a*_0_) of the as-synthesized sample based on SAXS data is as large as 40.3 nm. After calcinations, OMCs-7 exhibits at least three peaks with reciprocal *d*-spacing values following the relationship $\sqrt{3}:\sqrt{8}:\sqrt{11}$, which can be assigned to [111], [220], and [311] reflections of $\mathrm{F}\mathrm{d}\overline{3}\mathrm{m}$ space group combination with the TEM images in Fig. 3c, d [17]. The lattice parameter (*a*_0_) of OMCs-7 is 27.2 nm, which has a framework shrinkage of 32.5 %. We succeeded in recording TEM images along the (211) and (110) directions, which further revealed regular and extended pore periodicity. The formation of fascinating cubic $\mathrm{F}\mathrm{d}\overline{3}\mathrm{m}$ mesostructure might be due to the typical phase behaviors of the reverse copolymer adopted in our experimental condition. There exist sixteen small pores and eight larger pores in one unit cell on a double diamond network. With the ratio of 6/14, 8/12, and 9/11, the corresponding samples OMCs-6, OMCs-8, and OMCs-9 also have the $\mathrm{F}\mathrm{d}\overline{3}\mathrm{m}$ space group. Their SAXS patterns are shown in Additional file 1: Figures S2, S3, and S4, and some representative TEM images of OMCs-9 along different directions can be seen in Additional file 1: Figure S5 (ESI).

Figure 2c presents the SAXS patterns of the samples before and after calcinations synthesized in the ratio 11/9, the calcined sample shows at least two peaks, which can be assigned to [111] and [200] reflections of Fm3m space group combination with the TEM images of OMCs-11 viewed from [211] and [110] directions of Fm3m in Fig. 3e, f [14, 31]. The uniform and spherical mesopores are clearly visible in these images. The Fm3m mesophase also can be obtained with the ratio 10/10, and the SAXS patterns and TEM image of OMCs-10 are demonstrated in Additional file 1: Figures S6 and S7, respectively (ESI).

Figure 2d gives the SAXS patterns of the samples synthesized in further ratio of 12/8. Intriguingly, the calcined sample OMCs-12 shows at least four peaks which can be indexed as [200], [210], [211], and [220] reflections of Pm3n group [24]. This is the first time that OMCs with Pm3n symmetry has been reported to the best of our knowledge. This is further confirmed by TEM images viewed from [210] (g) and [100] (h) directions of the Pm3n structure. It is noticeable that when the ratio went on increasing, the copolymer could not well dissolve in the mixed solvents. In this case, the EISA process was destroyed to some degree and the Fd3m symmetry could be obtained from the ratios 13/7, 14/6, and 15/5 indicating that the structure is the most stable when more water is bounded to the copolymer. The SAXS patterns and some representative TEM images are shown in Additional file 1: Figures S8, S9, and S10 (ESI).

**Fig. 4:**
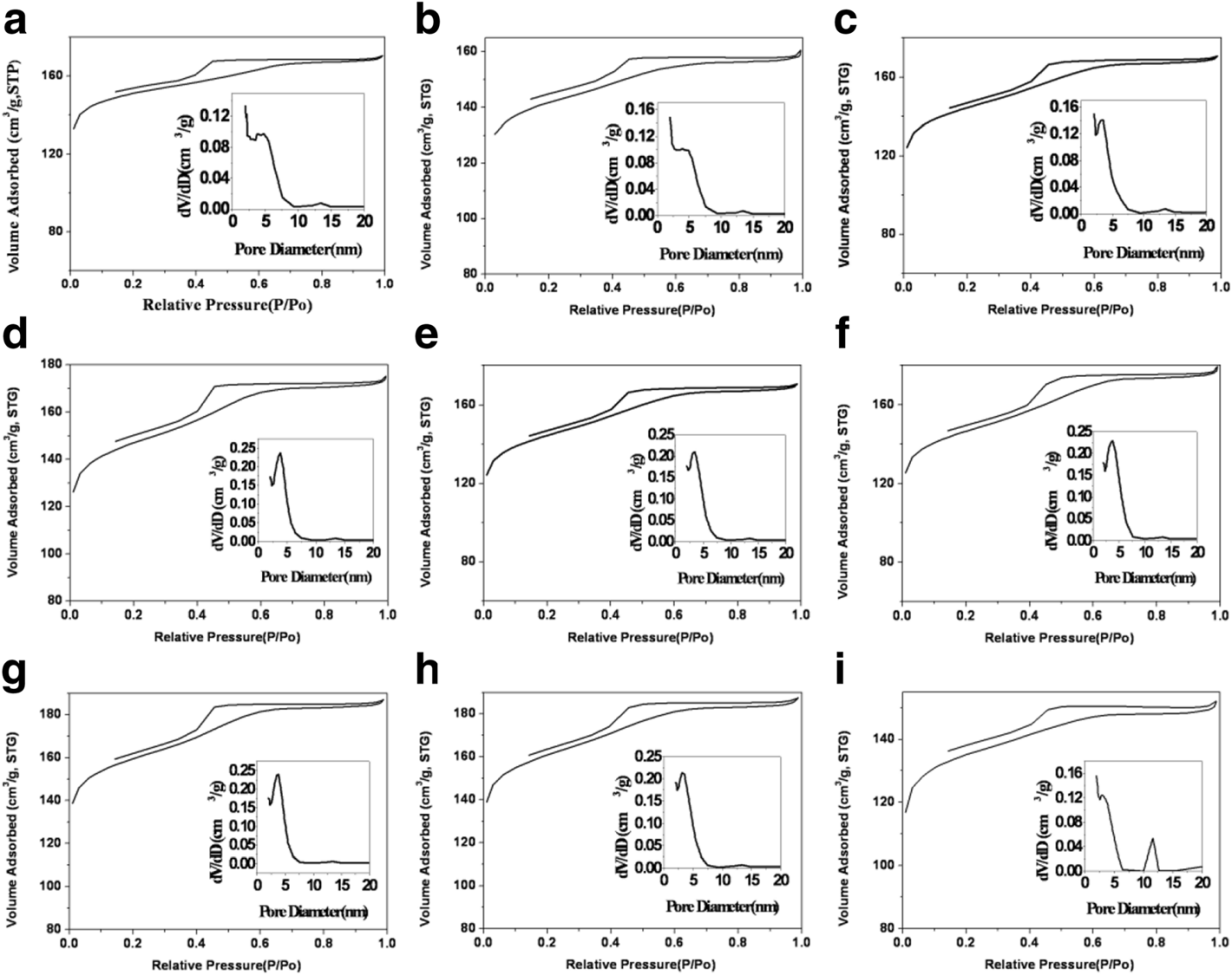
Nitrogen adsorption-desorption isotherms with corresponding pore size distributions (*inset*) of OMCs-6 (**a**), OMCs-7 (**b**), OMCs-8 (**c**), OMCs-9 (**d**), OMCs-11 (**e**), OMCs-12 (**f**), OMCs-13 (**g**), OMCs-14 (**h**), and OMCs-15 (**i**)

Nitrogen adsorption-desorption isotherms for the carbonized samples OMCs-*X* (*X* = 6, 7, 8, 9, 11, 12, 13, 14, and 15, respectively) show type IV reversible isotherm curves indicating that all OMCs are typical mesoporous materials, and their corresponding BJH pore size distributions are inset Fig. 4. Table 1 shows the parameters of typical OMCs-X; it can be seen that the obtained OMCs-*X* have similar surface area and pore volume. OMCs-5 has the highest mesopore ratio owing to the straight pore of 2-D hexagonal mesostructure.

**Table 1:** Pore parameters of typical OMCs-*X*

Sample	*S*_BET_ (m^2^/g)	*V*_total_ (cm^3^/g)	*V*_micro_ (cm^3^/g)	*V*_meso_ (cm^3^/g)	Ratio_meso_ (%)
OMCs-5	503	0.34	0.14	0.20	59
OMCs-8	478	0.25	0.18	0.07	28
OMCs-11	496	0.27	0.17	0.10	37
OMCs-15	455	0.25	0.17	0.08	32

Ratio_meso_ = (*V*_total_ − *V*_micro_)/*V*_total_

For comparison, we further investigated the effects of the cosolvents on the synthesis of OMCs directed by normal copolymer F127 as template. Our results demonstrated that no matter how the ratios of water/ethanol, hexane/ethanol, and heptane/ethanol were tuned, the mesophase kept unvaried (see Additional file 1: Figures S11, S12, and S13), suggesting that both the reverse copolymer and cosolvent water play pivotal roles in the mesophase transitions of OMCs which cannot take place when the normal copolymer F127 was used.

**Fig. 5:**
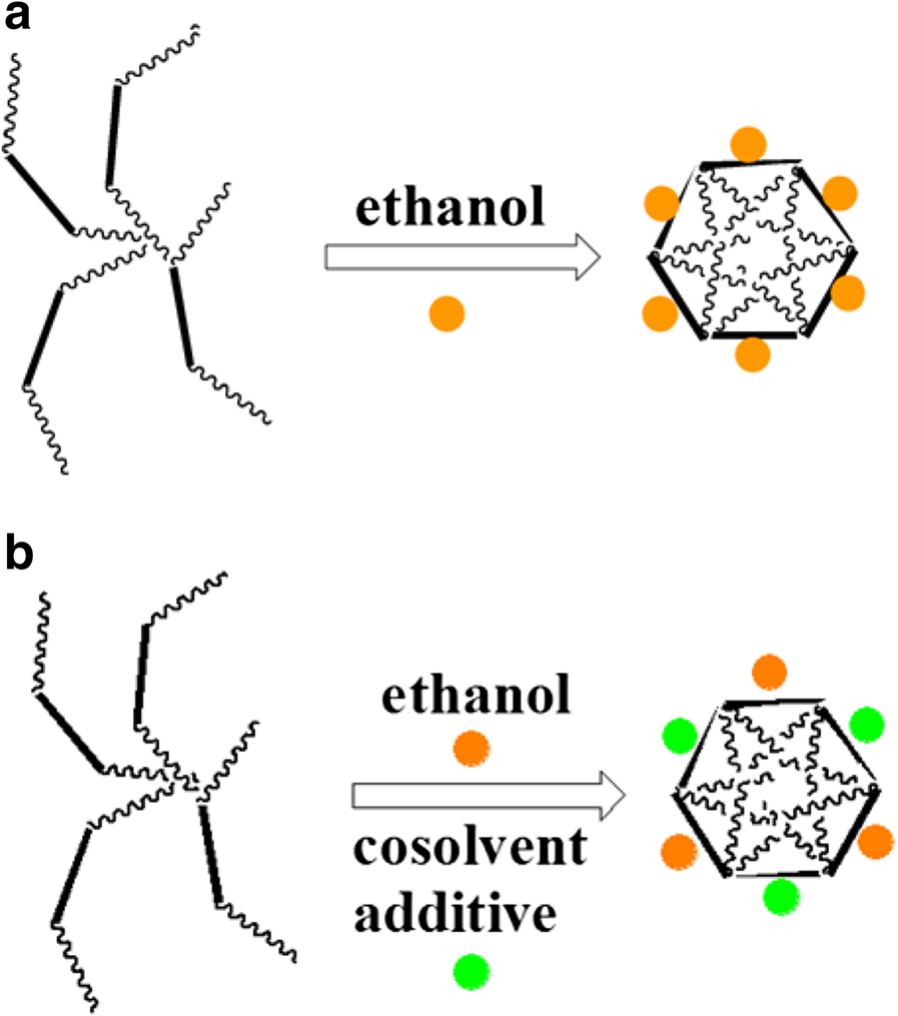
Micelle without and with water as cosolvent (**a**, **b**)

The effects of varied ratios of water/ethanol on the formation of mesostructures can be explained by the interfacial interaction of different bonding solvents along the PEO part as shown in Fig. 5. When the cosolvent is free (as demonstrated in Fig. 5a), only the solvent ethanol molecules have interaction with the PEO part, and the p6mm mesophase can be formed under our experimental condition. However, as the cosolvent water takes part in the micelle, the parameter varies because of the different polarity of water and ethanol (as demonstrated in Fig. 5a). As the amount of water increases, the *a* value increases in virtue of increased polarity of the solution. In the present reaction or system, varied ratios of water/ethanol will enable more or less water molecules run into the PEO part of the surfactant. The copolymer which bounded less water molecules will result in the hexagonal mesophase with a large *g* value and low curvatures; however, copolymer with more bounded water will result in cubic mesophase with a low *a* value and high curvature.

## Conclusions

In a word, varied mesophases of OMCs have been achieved directly utilizing a homemade reverse copolymer as a soft template and low molecular resin as a precursor by an EISA method as the hydration increases. In the experiment, the water content of the mixed solvents water/ethanol plays key roles in the mesophase transformation. There have been four types of well-ordered mesophases, 2-D hexagonal p6mm, 3-D cubic $\mathrm{F}\mathrm{d}\overline{3}\mathrm{m}$, $\mathrm{F}\mathrm{m}\overline{3}\mathrm{m}$, and Pm3n, which can be synthesized in the presence of varied water contents of mixed water/ethanol while keeping the surfactant and carbon precursor constant. These changes of pore structure show a dramatic mesopore geometry change with an increase of mesophase interface curvature as the amount of water increases. The synthesis of OMCs with varied symmetries will promote us to understand the mechanism of mesopore formation and supply a potential method to synthesize new mesophase of OMCs.

## Additional file

Additional file 1:
**Figure S1.** 1H-NMR (300MHz, CDCl3) spectrum of triblock copolymer PO97EO186PO97. **Figure S2.** SAXS patterns of OMCs-6 a: as made; b calcined sample. **Figure S3.** SAXS patterns of OMCs-8 a: as made; b calcined sample. **Figure S4.** SAXS patterns of OMCs-9 calcined sample. **Figure S5.** TEM images of OMCs-9, viewed from [110] (a), [100] (b) and [111] directions. The insets are the corresponding FFT diffractograms. **Figure S6.** SAXS patterns of OMCs-10 a: as made; b calcined sample. **Figure S7.** TEM image of OMCs-10 viewed from [211] direction. **Figure S7.** SAXS patterns of OMCs-13 a: as made; b calcined sample. **Figure S8.** SAXS patterns of OMCs-14 a: as made; b calcined sample. **Figure S9.** TEM image of OMCs-14 viewed from [110] direction with different magnifications. **Figure S10.** TEM image of OMCs-15 viewed from [100] direction with different magnifications. **Figure S11.** The effects of varied amounts of water on the structure of OMC. **Figure S12.** The effects of varied amounts of hexane on the structure of OMC. **Figure S13.** The effects of varied amounts of heptane on the structure of OMC. (DOC 1.98 MB)
