# Supplementary material for: Structure Evolution of Ordered Mesoporous Carbons Induced by Water Content of Mixed Solvents Water/Ethanol
Source: Nanoscale Res Lett. 2016 Aug 12;11:361. doi: 10.1186/s11671-016-1569-4 (PMC4987573; doi:10.1186/s11671-016-1569-4)
Supplement: Additional file 1: — Figure S1. 1H-NMR (300MHz, CDCl3) spectrum of triblock copolymer PO97EO186PO97. Figure S2. SAXS patterns of OMCs-6 a: as made; b calcined sample. Figure S3. SAXS patterns of OMCs-8 a: as made; b calcined sample. Figure S4. SAXS patterns of OMCs-9 calcined sample. Figure S5. TEM images of OMCs-9, viewed from [110] (a), [100] (b) and [111] directions. The insets are the corresponding FFT diffractograms. Figure S6. SAXS patterns of OMCs-10 a: as made; b calcined sample. Figure S7. TEM image of OMCs-10 viewed from [211] direction. Figure S7. SAXS patterns of OMCs-13 a: as made; b calcined sample. Figure S8. SAXS patterns of OMCs-14 a: as made; b calcined sample. Figure S9. TEM image of OMCs-14 viewed from [110] direction with different magnifications. Figure S10. TEM image of OMCs-15 viewed from [100] direction with different magnifications. Figure S11. The effects of varied amounts of water on the structure of OMC. Figure S12. The effects of varied amounts of hexane on the structure of OMC. Figure S13. The effects of varied amounts of heptane on the structure of OMC. (DOC 1.98 MB) [file 11671_2016_1569_MOESM1_ESM.doc]

Structure Evolution of Ordered Mesoporous Carbons Induced by Water

Content of Mixed Solvents Water/Ethanol

Peng Li^a^, Shujun Lianga[[1]](#footnote-2)*, Zhenzhong Li^a^, Yan Zhai^a^, Yan Songb[[2]](#footnote-3)*

a Department of Materials Engineering, Taiyuan Institute of Technology, Taiyuan 030008, China

b Key Laboratory of Carbon Materials, Institute of Coal Chemistry, Taiyuan 030001, China

*E-mail:*  *lshj7825@163.com (S. Liang), yansong1026@163.com*

**
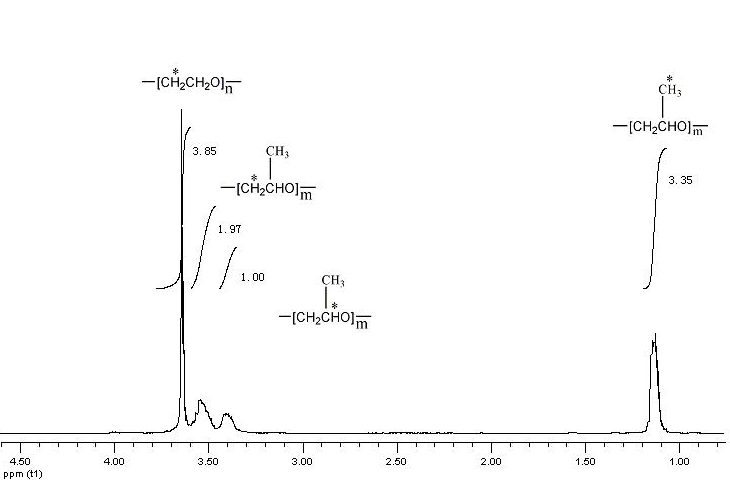
**

**Chemical shift ppm**

**Fig. S1** 1H-NMR (300MHz, CDCl_3_) spectrum of triblock copolymer PO_97_EO_186_PO_97_.


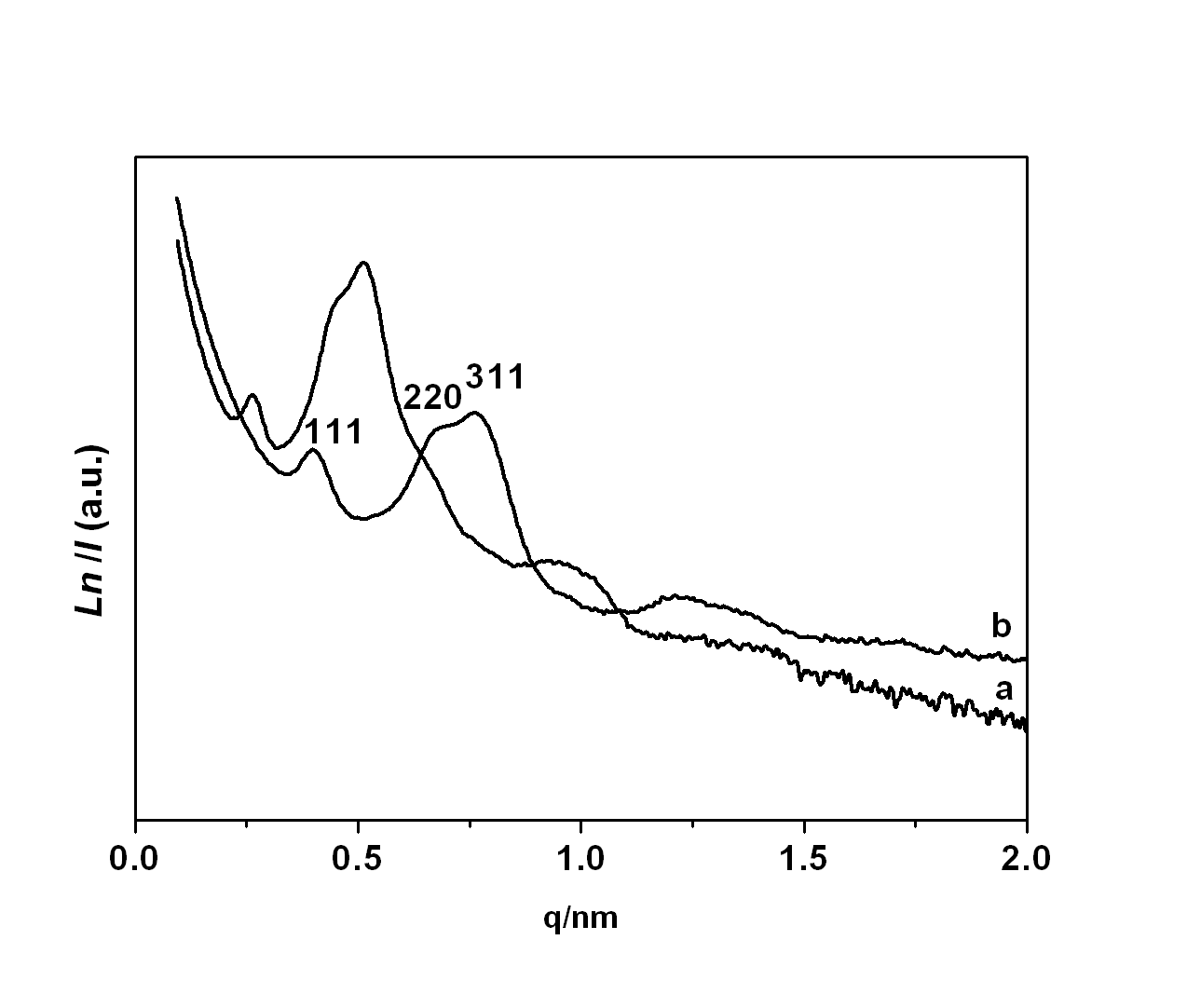


**Fig. S2** SAXS patterns of OMCs-6 a: as made; b calcined sample


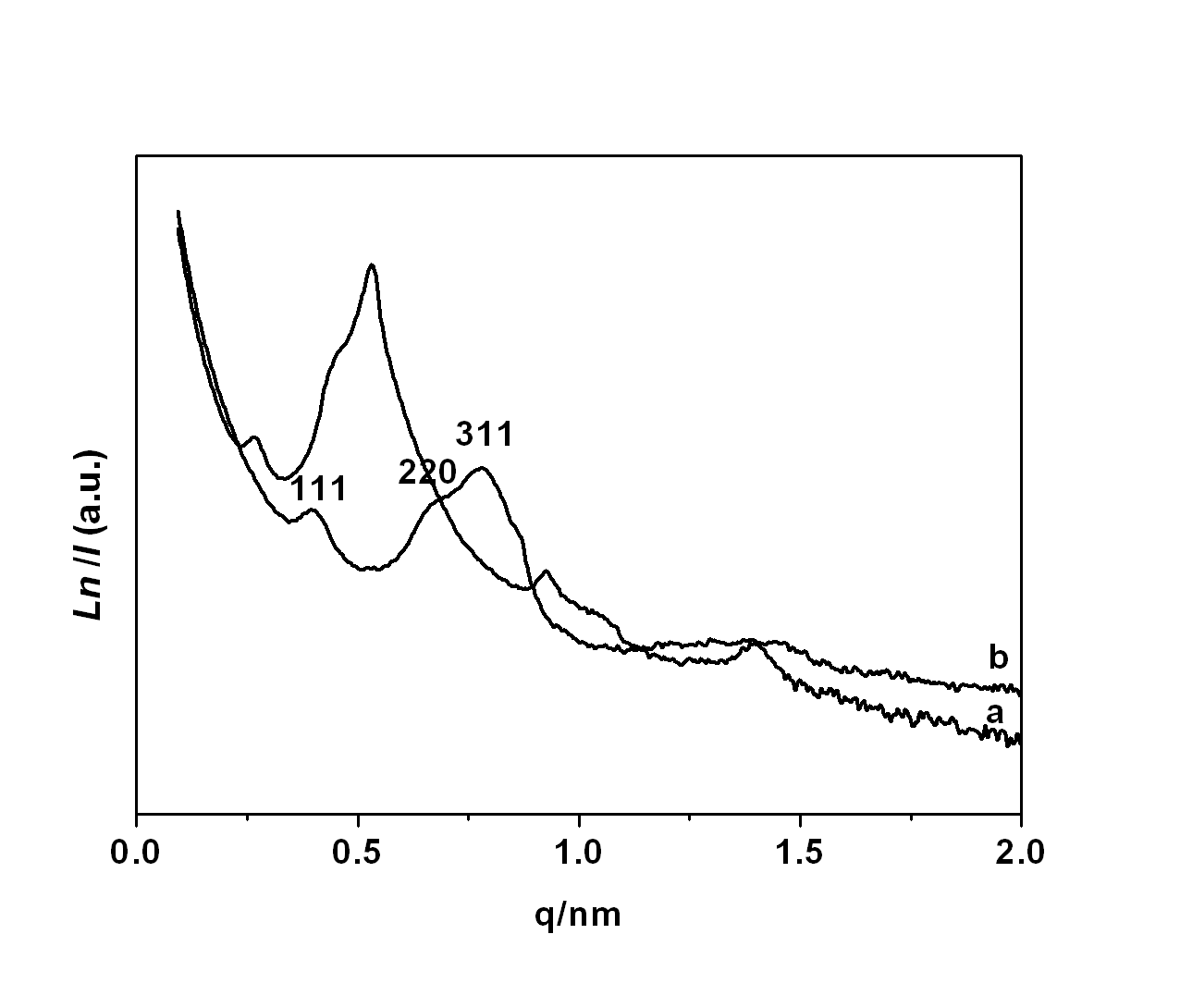


**Fig. S3** SAXS patterns of OMCs-8 a: as made; b calcined sample


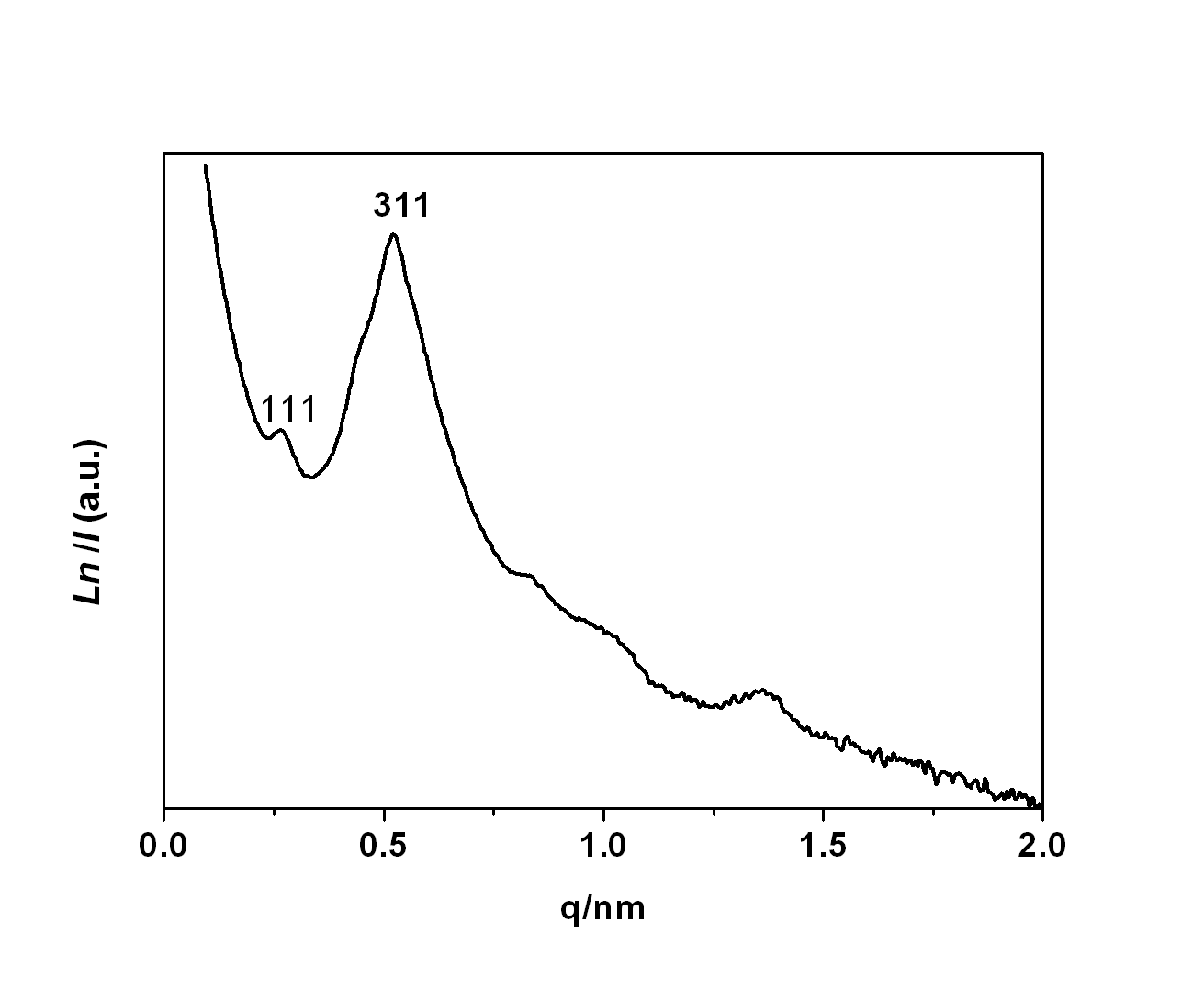


**Fig. S4** SAXS patterns of OMCs-9 calcined sample

**
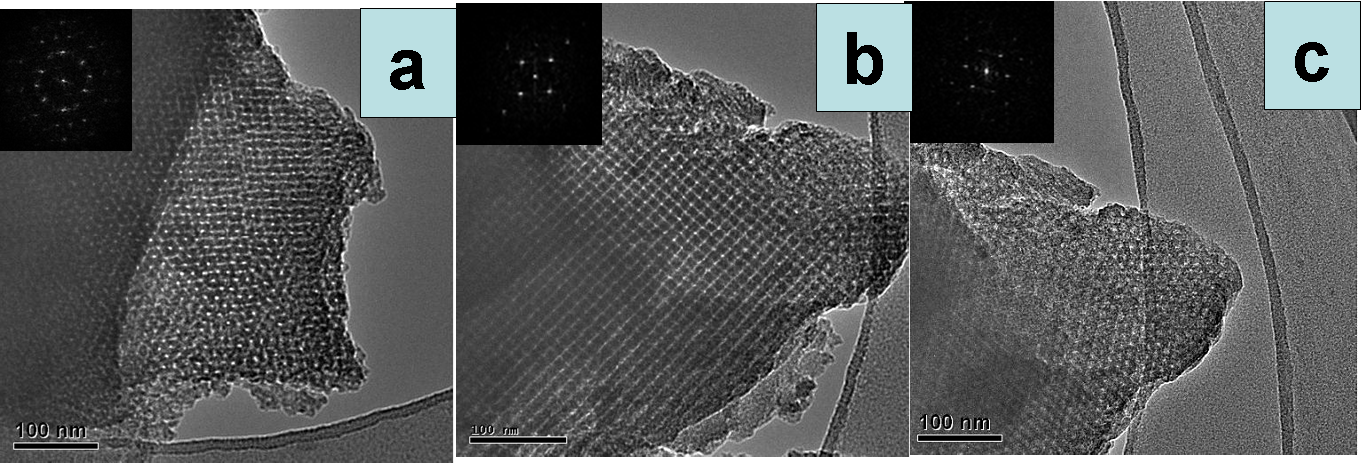
**

**Fig. S5** TEM images of OMCs-9, viewed from [110] (a), [100] (b) and [111] directions. The insets are the corresponding FFT diffractograms


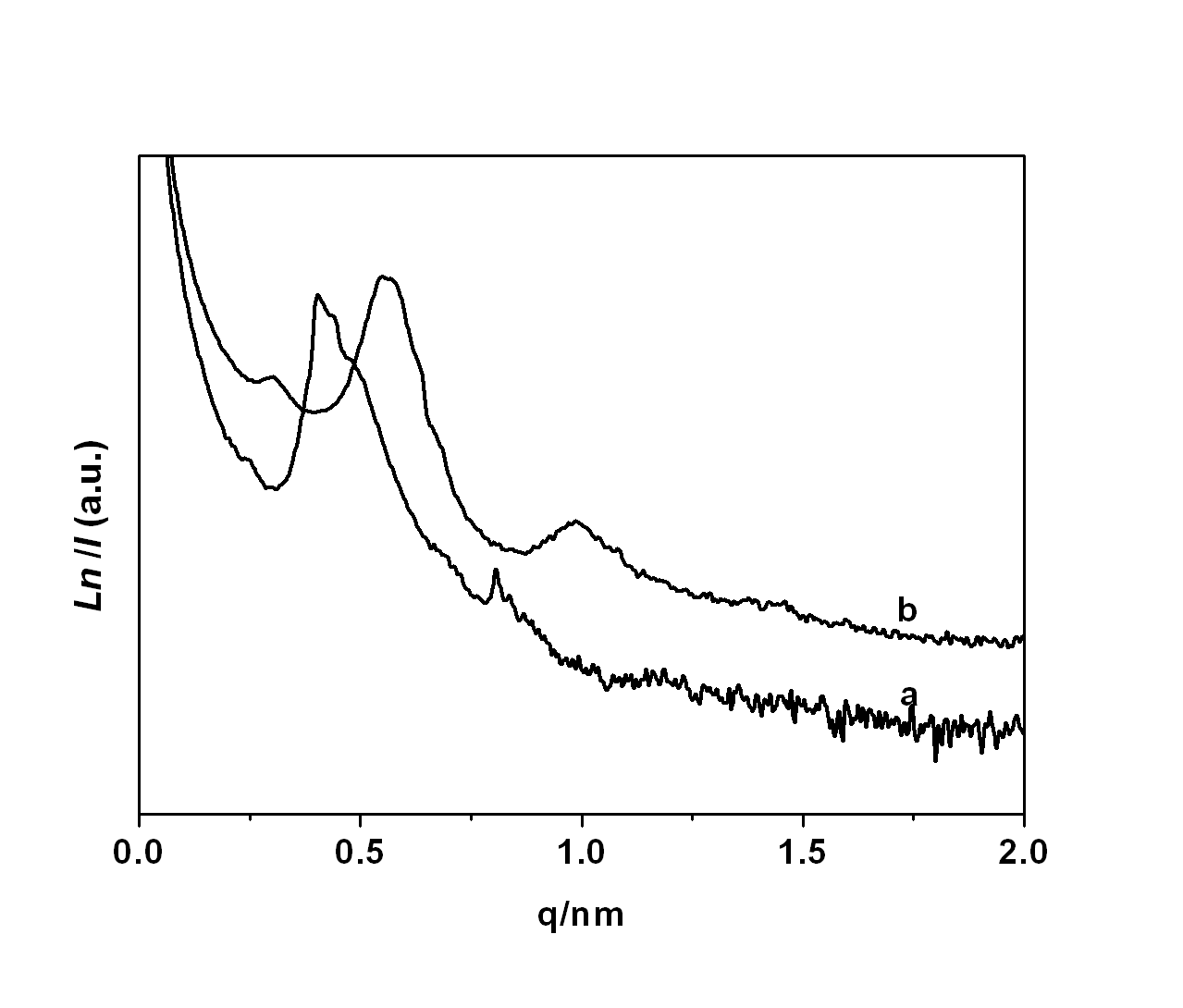


**Fig. S6** SAXS patterns of OMCs-10 a: as made; b calcined sample


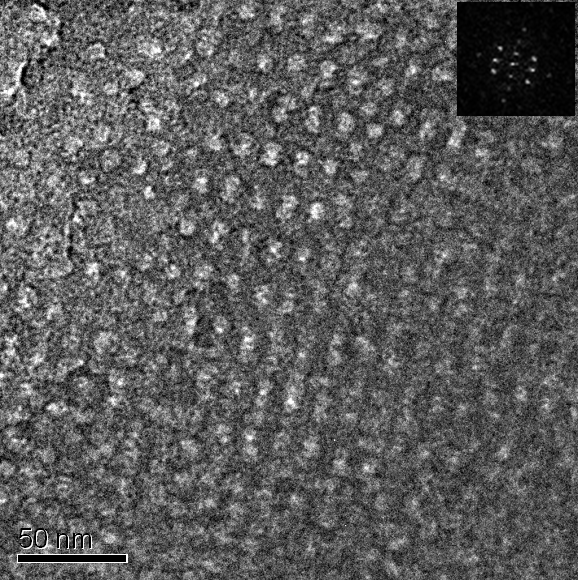


**Fig. S7** TEM image of OMCs-10 viewed from [211] direction


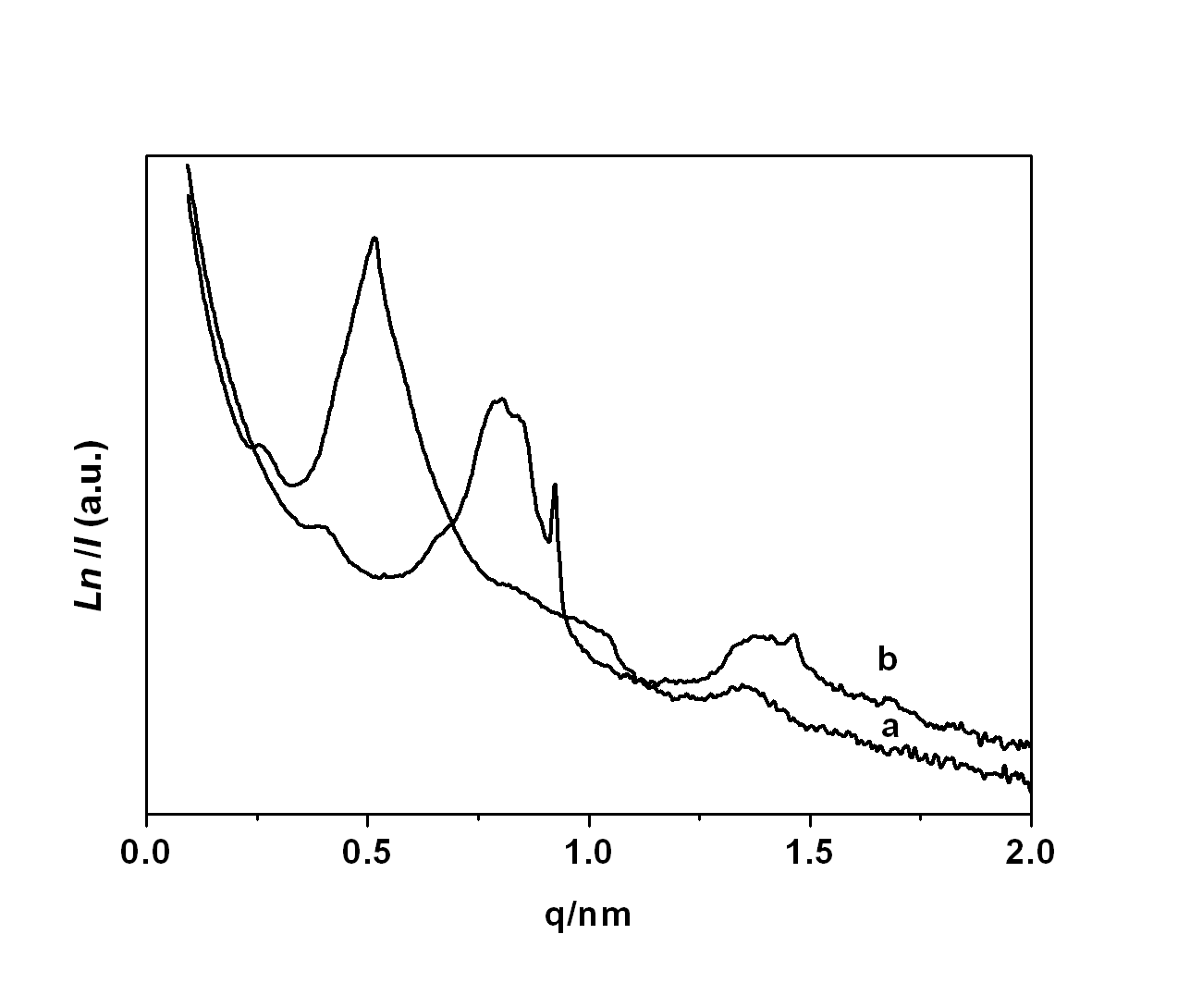


**Fig. S7** SAXS patterns of OMCs-13 a: as made; b calcined sample


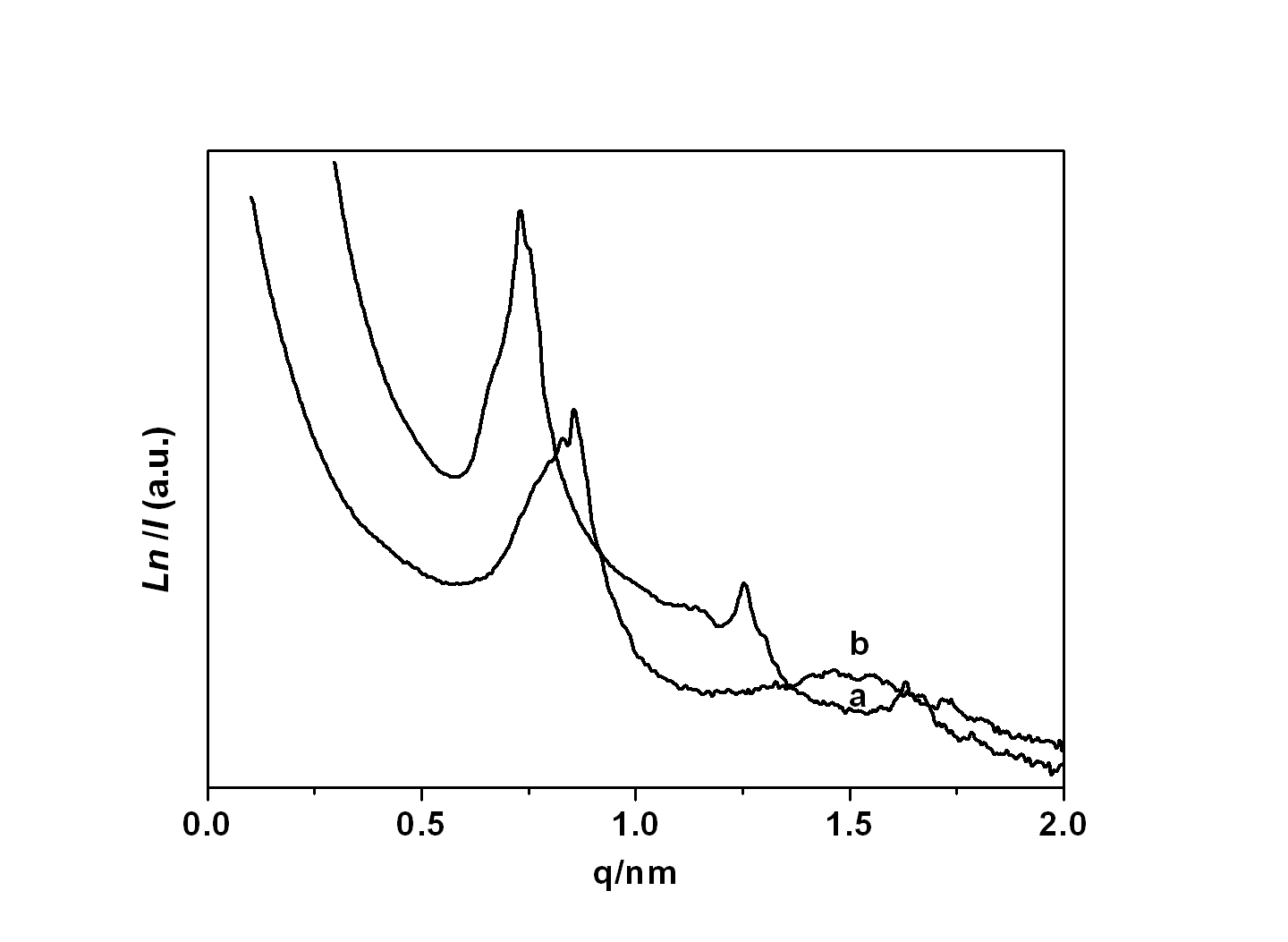


**Fig. S8** SAXS patterns of OMCs-14 a: as made; b calcined sample


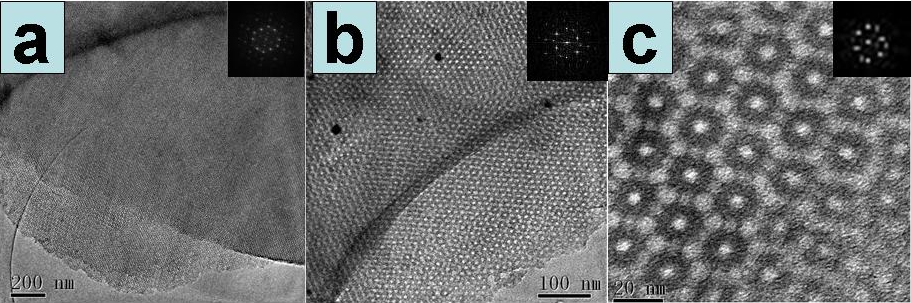


**Fig. S9** TEM image of OMCs-14 viewed from [110] direction with different magnifications

**
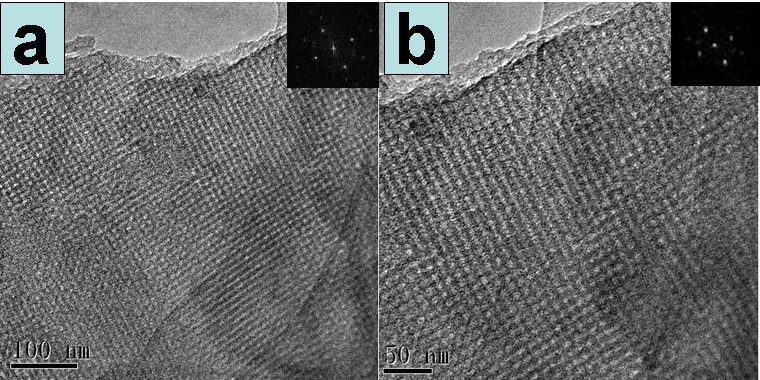
**

**Fig. S10** TEM image of OMCs-15 viewed from [100] direction with different magnifications


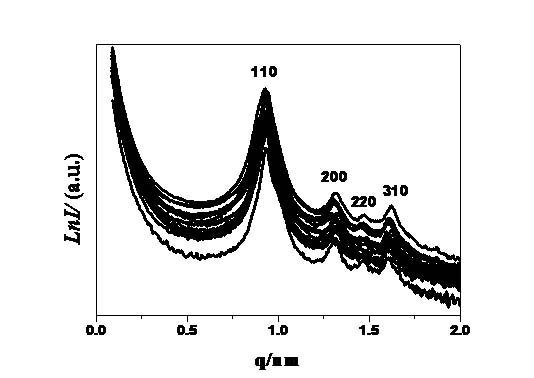


**Fig. S11 The effects of varied amounts of water on the structure of OMC**

**
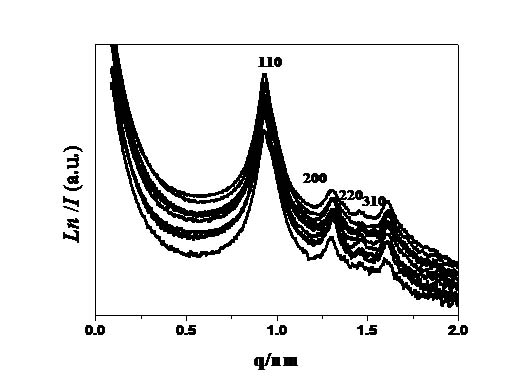
**

**Fig. S12 The effects of varied amounts of hexane on the structure of OMC**

**
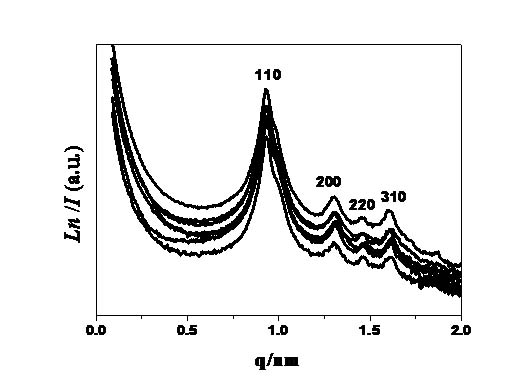
**

**Fig. S13 The effects of varied amounts of heptane on the structure of OMC**

1. ^*^Corresponding author. Tel.: Fax: +86 351 3569624. E-mail address: lshj7825@163.com

   (S. Liang), [yansong1026@126.com(Y](mailto:yansong1026@126.com(Y). Song). [↑](#footnote-ref-2)
2. ^*^ [↑](#footnote-ref-3)
